# Supplementary material for: Increased Glycine-N-methyltransferase expression disrupts light-dependent gene expression rhythms in the Drosophila eye
Source: J Cell Sci. 2026 Apr 17;139(7):jcs264529. doi: 10.1242/jcs.264529 (PMC13120682; doi:10.1242/jcs.264529)
Supplement: Supplementary information [file joces-139-264529-s1.pdf]

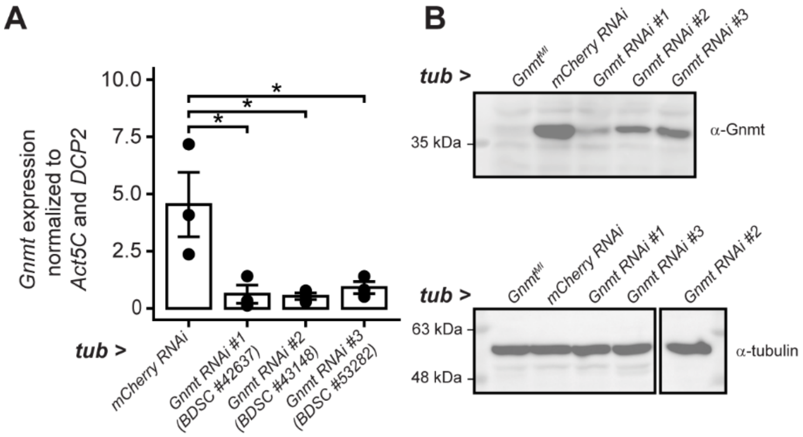

Fig. S1. qPCR and western blotting validation of Gnmt RNAi. a, qRT-PCR showing expression in indicated RNAi lines under *tub-Gal4* control. Relative mRNA levels (expression) were calculated as the geometric mean of starting quantity normalized to Actin 5C (*Act5C*) and Decapping protein 2 (*Dcp2*). Gnmt RNAi #1 – BDSC:42637, RNAi #2 – BDSC:43148, RNAi #3 – BDSC:53282. Graph depicts mean ± s.d. with individual replicates overlaid as points (*n*=3). *p*-value (* < 0.05), one-way ANOVA with Tukey's post hoc test. **b**, Western blot of Gnmt in larvae. Tubulin is shown as a loading control on separate blots. *Gnmt*^{MI} – BDSC:67643 is a mutant that lacks Gnmt expression.

Journal of Cell Science • Supplementary information

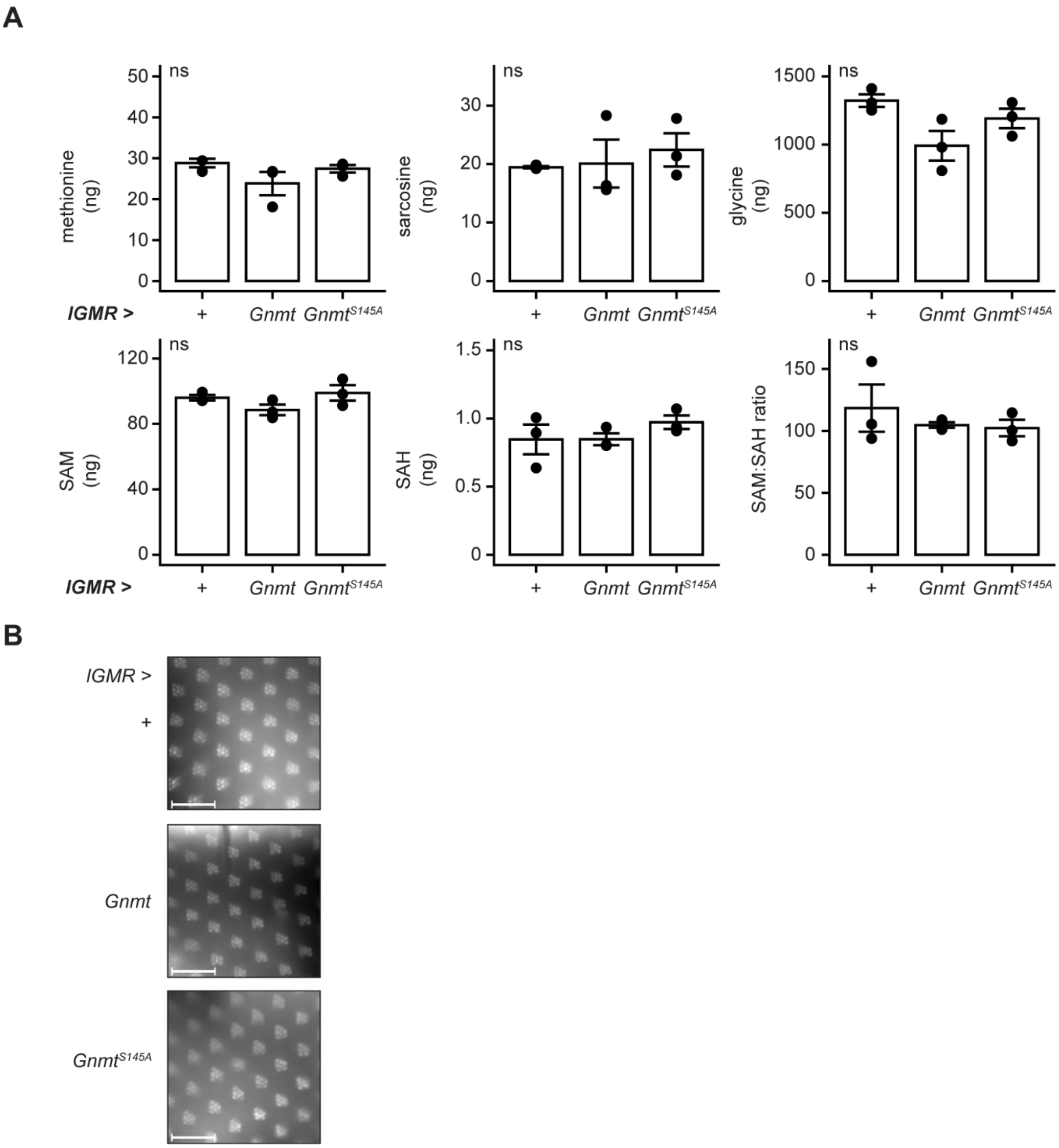

Fig. S2. Eye-specific overexpression of Gnmt does not influence whole head metabolite levels or retinal degeneration. a, LC-MS/MS analysis of SAM, SAH, methionine, glycine, and sarcosine levels in heads of D10 adult male flies overexpressing Gnmt or Gnmt^{S145A} under *IGMR-Gal4* control. Driver outcrossed to w^{1118} (+) was used as control. Graph depicts mean ± s.d. with individual replicates overlaid as points (*n*=3). *p*-value (ns > 0.05) one-way ANOVA. **b**, representative images of optic neutralization. Scale bar = 25 μm.

Journal of Cell Science • Supplementary information

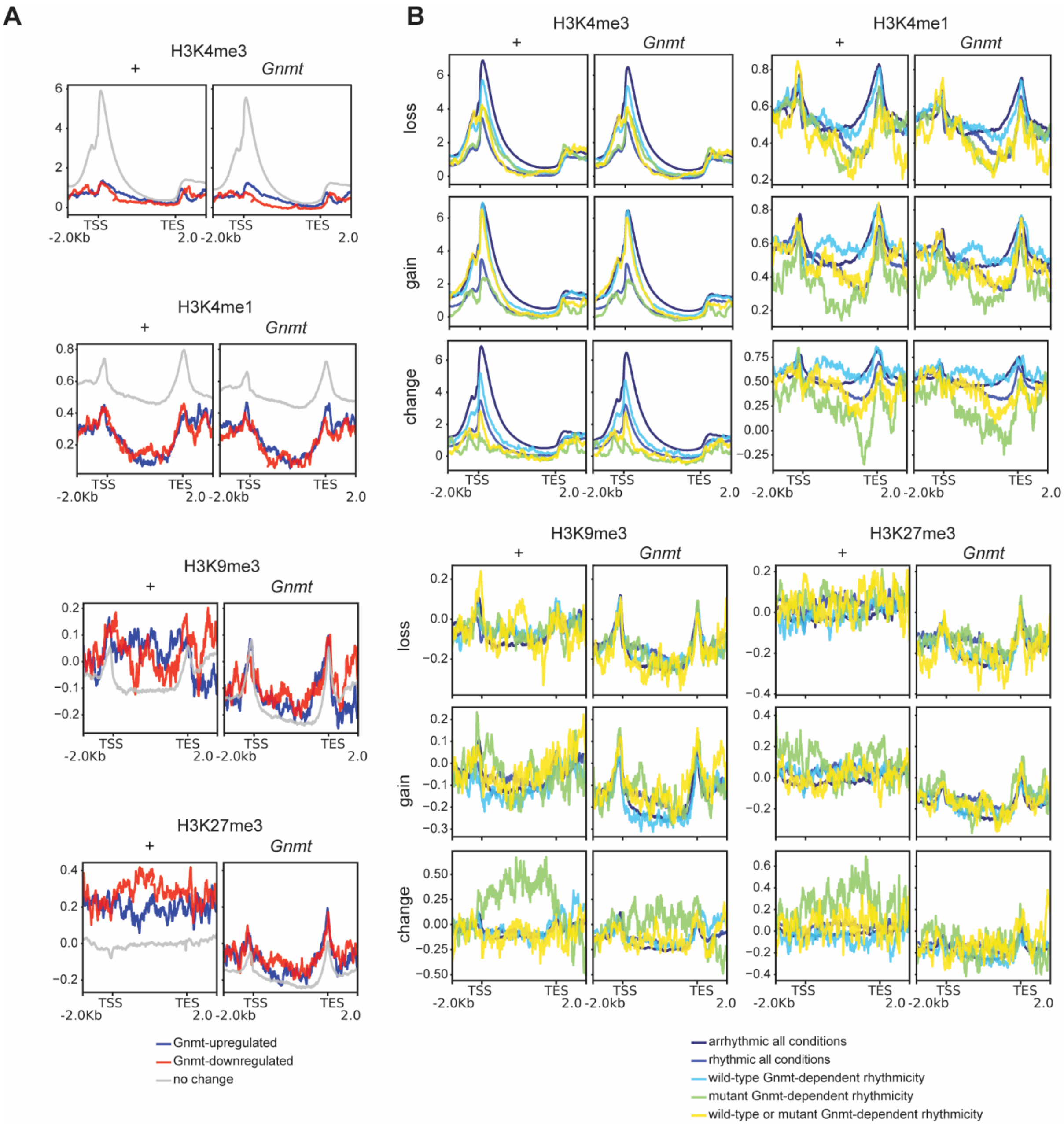

Fig. S3. Eye-specific overexpression of Gnmt decreases repressive histone methylation across all gene expression categories. a, Gene metaplots representing mean normalized counts for differentially expressed genes. Metaplots depict transcription start site (TSS) and transcription end site (TES) ± 2.0 kb with gene bodies scaled to 5.0 kb. **b**, Gene metaplots representing mean normalized counts for differential rhythmicity categories.

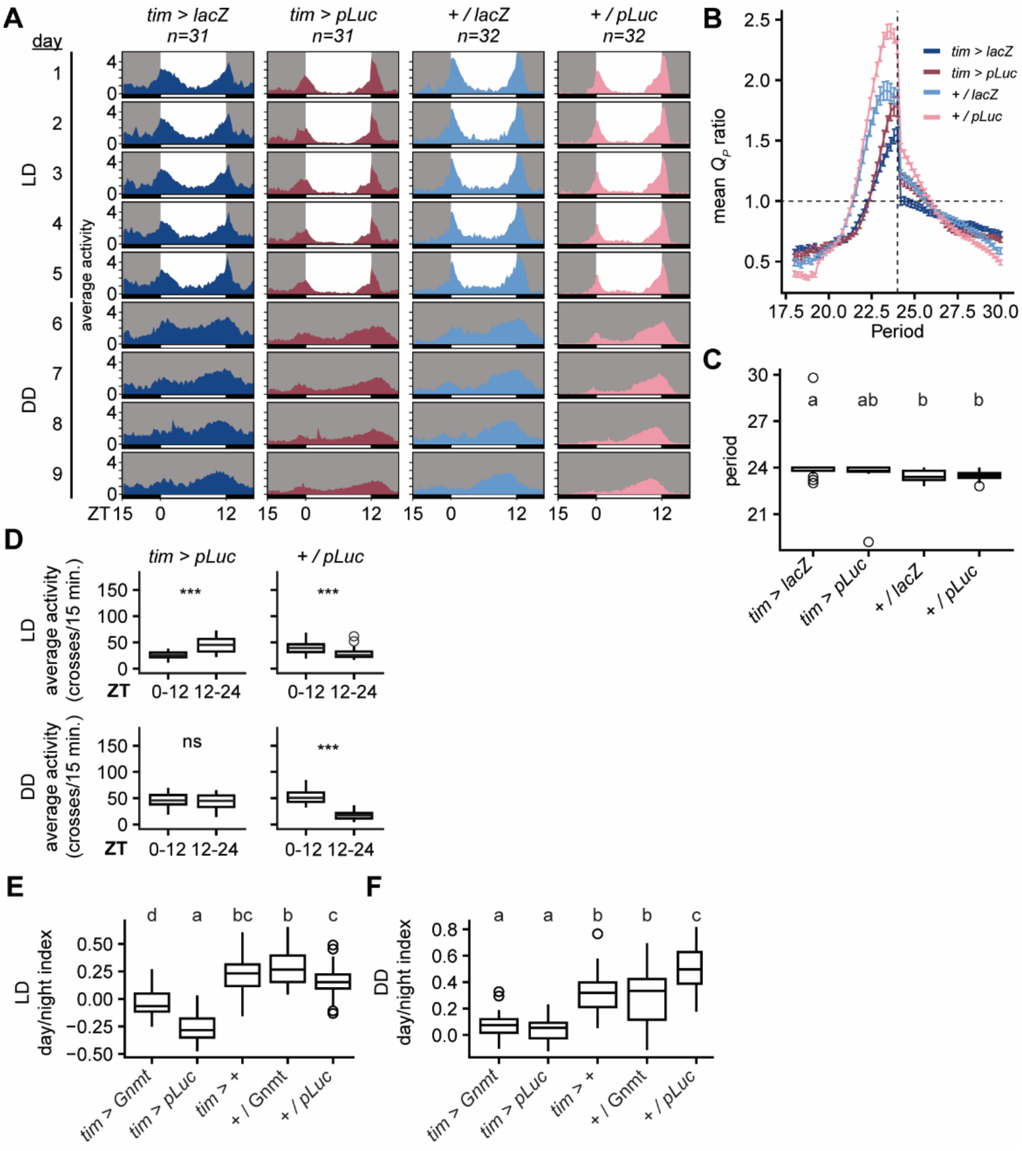

Fig. S4. Additional circadian behavior assay controls. A, Actograms showing averaged activity in flies of the indicated genotypes during light:dark (LD) and dark:dark (DD) conditions. Background color denotes status of the light (white = on, grey = off). Horizontal bar denotes subjective day (white) and night (black) based on ZT. (+) indicates lines crossed with w^{1118}. *n* indicated above each plot. *lacZ* – β-galactosidase, *pLuc* – firefly luciferase. **B**, Chi-square circadian periodogram analysis of rhythmic locomotor activity under DD conditions. The ratio of observed periodogram power to the significance threshold is plotted as a function of the tested rhythmic period: (Q_P ratio = Q_P activity / Q_P significance). Period testing range = 18-30 hrs with a testing resolution of 0.2 hrs. Error bars depict mean ± standard error of the mean (s.e.m.). Mean Q_P ratio above 1 denotes a significant circadian period. Vertical dashed line corresponds with a period of 24-hours. **C,** boxplots depicting circadian periods of individual flies under DD conditions. Different letters indicate statistical significance where *p*-value (< 0.05), one-way ANOVA. **D,** boxplots depicting average activity (crosses/15 minutes) of individual flies in either LD or DD conditions. Data are binned into either Day (ZT 0-12) or Night (ZT 12-24). *p-value* (ns > 0.05, *** < 0.001), Wilcoxon signed-rank test. **E,F,** boxplots depicting Day/night index of individual flies in either LD or DD conditions. Different letters indicate statistical significance where *p*-value (< 0.05), one-way ANOVA with Tukey's multiple comparisons post hoc test.

Journal of Cell Science • Supplementary information

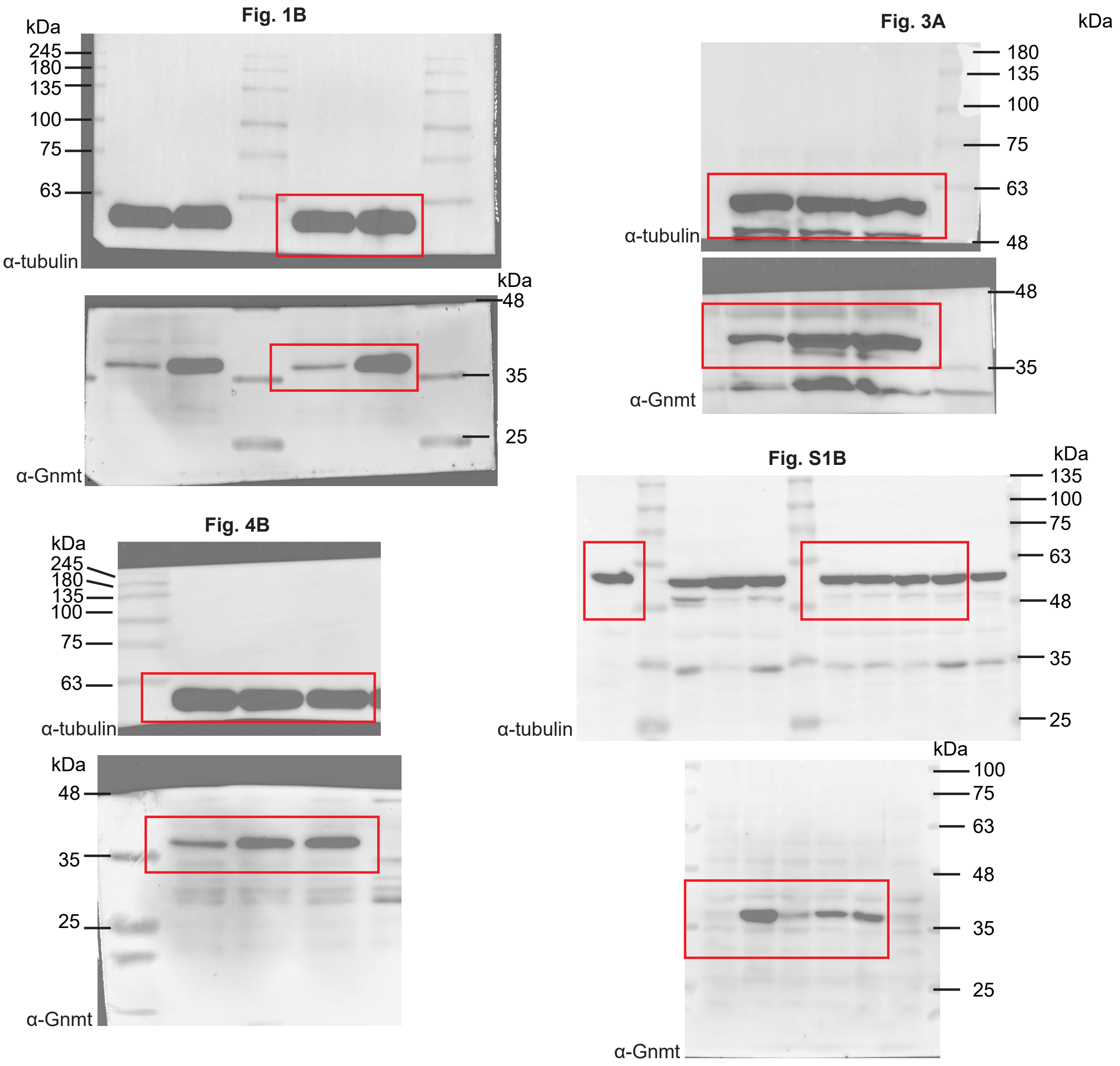

Fig. S5. Blot transparency

Journal of Cell Science • Supplementary information

Table S1. Drosophila Stocks

Available for download at
https://journals.biologists.com/jcs/article-lookup/doi/10.1242/jcs.264529#supplementary-data

Table S2. Primer Sequences

Available for download at
https://journals.biologists.com/jcs/article-lookup/doi/10.1242/jcs.264529#supplementary-data

Table S3. dryR Results

Available for download at
https://journals.biologists.com/jcs/article-lookup/doi/10.1242/jcs.264529#supplementary-data

Table S4. Differential Gene Expression (DEseq2) lGMR > Gnmt vs. lGMR > +

Available for download at
https://journals.biologists.com/jcs/article-lookup/doi/10.1242/jcs.264529#supplementary-data

Table S5. Differential Gene Expression (DEseq2) lGMR > GnmtS145A vs. lGMR > +

Available for download at
https://journals.biologists.com/jcs/article-lookup/doi/10.1242/jcs.264529#supplementary-data

Table S6. CUT&RUN Peaks

Available for download at
https://journals.biologists.com/jcs/article-lookup/doi/10.1242/jcs.264529#supplementary-data

Table S7. LC-MS/MS Multiple reaction monitoring

Available for download at
https://journals.biologists.com/jcs/article-lookup/doi/10.1242/jcs.264529#supplementary-data

Journal of Cell Science • Supplementary information
